# Heparin resistance in severe thermal injury: a prospective cohort study

**DOI:** 10.1093/burnst/tkab032

**Published:** 2021-10-20

**Authors:** Liam D Cato, Benjamin Bailiff, Joshua Price, Christos Ermogeneous, Jon Hazeldine, William Lester, Gillian Lowe, Christopher Wearn, Jonathan R B Bishop, Janet M Lord, Naiem Moiemen, Paul Harrison

**Affiliations:** Scar Free Foundation Birmingham Centre for Burns Research, University Hospitals Birmingham Foundation Trust, Queen Elizabeth Hospital Birmingham, Mindelsohn Way, Birmingham, B15 2WB, UK; Institute of Inflammation and Ageing, University of Birmingham, Birmingham, B15 2TT, UK; Department of Haematology, University Hospitals Birmingham Foundation Trust, Mindelsohn Way, Birmingham, B15 2WB, UK; Institute of Inflammation and Ageing, University of Birmingham, Birmingham, B15 2TT, UK; Institute of Inflammation and Ageing, University of Birmingham, Birmingham, B15 2TT, UK; Institute of Inflammation and Ageing, University of Birmingham, Birmingham, B15 2TT, UK; Department of Haematology, University Hospitals Birmingham Foundation Trust, Mindelsohn Way, Birmingham, B15 2WB, UK; Department of Haematology, University Hospitals Birmingham Foundation Trust, Mindelsohn Way, Birmingham, B15 2WB, UK; Scar Free Foundation Birmingham Centre for Burns Research, University Hospitals Birmingham Foundation Trust, Queen Elizabeth Hospital Birmingham, Mindelsohn Way, Birmingham, B15 2WB, UK; Institute of Inflammation and Ageing, University of Birmingham, Birmingham, B15 2TT, UK; NIHR Surgical Reconstruction and Microbiology Research Centre, University Hospitals Birmingham Foundation Trust, Mindelsohn Way, Birmingham, B15 2WB, UK; Scar Free Foundation Birmingham Centre for Burns Research, University Hospitals Birmingham Foundation Trust, Queen Elizabeth Hospital Birmingham, Mindelsohn Way, Birmingham, B15 2WB, UK; Institute of Inflammation and Ageing, University of Birmingham, Birmingham, B15 2TT, UK; NIHR Surgical Reconstruction and Microbiology Research Centre, University Hospitals Birmingham Foundation Trust, Mindelsohn Way, Birmingham, B15 2WB, UK; Scar Free Foundation Birmingham Centre for Burns Research, University Hospitals Birmingham Foundation Trust, Queen Elizabeth Hospital Birmingham, Mindelsohn Way, Birmingham, B15 2WB, UK; Institute of Inflammation and Ageing, University of Birmingham, Birmingham, B15 2TT, UK; NIHR Surgical Reconstruction and Microbiology Research Centre, University Hospitals Birmingham Foundation Trust, Mindelsohn Way, Birmingham, B15 2WB, UK; Scar Free Foundation Birmingham Centre for Burns Research, University Hospitals Birmingham Foundation Trust, Queen Elizabeth Hospital Birmingham, Mindelsohn Way, Birmingham, B15 2WB, UK; Institute of Inflammation and Ageing, University of Birmingham, Birmingham, B15 2TT, UK; NIHR Surgical Reconstruction and Microbiology Research Centre, University Hospitals Birmingham Foundation Trust, Mindelsohn Way, Birmingham, B15 2WB, UK

**Keywords:** Burn, Heparin resistance, Low molecular-weight heparin, Nucleosomes, Neutrophil extracellular traps, Factor-Xa, Thrombosis, Enoxaparin, NETosis

## Abstract

**Background:**

Low molecular-weight heparin (LMWH) is routinely administered to burn patients for thromboprophylaxis. Some studies have reported heparin resistance, yet the mechanism(s) and prevalence have not been systematically studied. We hypothesized that nucleosomes, composed of histone structures with associated DNA released from injured tissue and activated immune cells in the form of neutrophil extracellular traps (NETs or NETosis), neutralize LMWH resulting in suboptimal anticoagulation, assessed by reduction in anti-factor Xa activity.

**Methods:**

Blood was sampled from >15% total body surface area (TBSA) burn patients receiving LMWH on days 5, 10 and 14. Peak anti-factor Xa (AFXa) activity, anti-thrombin (ATIII) activity, cell-free DNA (cfDNA) levels and nucleosome levels were measured. Mixed effects regression was adjusted for multiple confounders, including injury severity and ATIII activity, and was used to test the association between nucleosomes and AFXa.

**Results:**

A total of 30 patients with severe burns were included. Mean TBSA 43% (SD 17). Twenty-three (77%) patients were affected by heparin resistance (defined by AFXa activity <0.2 IU/mL). Mean peak AFXa activity across samples was 0.18 IU/mL (SD 0.11). Mean ATIII was 81.9% activity (SD 20.4). Samples taken at higher LWMH doses were found to have significantly increased AFXa activity, though the effect was not observed at all doses, at 8000 IU no samples were heparin resistant. Nucleosome levels were negatively correlated with AFXa (*r* = −0.29, *p* = 0.050) consistent with the hypothesis. The final model, with peak AFXa as the response variable, was adjusted for nucleosome levels (*p* = 0.0453), ATIII activity (*p* = 0.0053), LMWH dose pre-sample (*p* = 0.0049), drug given (enoxaparin or tinzaparin) (*p* = 0.03), and other confounders including severity of injury, age, gender, time point of sample.

**Conclusions:**

Heparin resistance is a prevalent issue in severe burns. Nucleosome levels were increased post-burn, and showed an inverse association with AFXa consistent with the hypothesis that they may interfere with the anticoagulant effect of heparin *in vivo* and contribute to heparin resistance. Accurate monitoring of AFXa activity with appropriate therapy escalation plans are recommended with dose adjustment following severe burn injury.

Highlights

- This is one of the first systematic prospective studies measuring heparin resistance in severe burn injury.
- Heparin resistance is highly prevalent among severe burn injury patients (>70%).
- We tested the hypothesis that nucleosomes from NETs and other sources may impair heparin’s anticoagulant ability.
- Nucleosome levels are elevated in severe burn patients and associated with a decrease in peak anti-factor Xa levels (a measure of low molecular-weight heparin anticoagulant ability).

## Background

Severe burn injury and subsequent surgery can promote haemostasis and hypercoagulation. Both thromboelastography and thrombin generation tests can identify hypercoagulability in burn patients upon admission [1–4]. Although haemostasis is beneficial for local early wound healing responses and prevention of blood loss, any systemic prothrombotic tendency can result in venous thromboembolism (VTE). Severe tissue damage following burn injury causes exposure of subendothelial tissue factor [5], whilst production of inflammatory cytokines as part of the systemic inflammatory response syndrome causes additional activation/priming of the clotting system. For example, levels of many clotting factors (e.g. fibrinogen, factor VIII and von Willebrand factor) increase in response to inflammation. Following stimulation, endothelial cells, monocytes and neutrophils release tissue factor into the systemic circulation [6]. Platelets are also significantly activated in burns resulting in the classical platelet count nadir at day 3 and subsequent high thrombopoietin levels with a resultant thrombocytosis [7–10]. In addition, neutrophil extracellular traps (NETs) released by neutrophils have been shown to be extensively released in severe burns and further activate both platelets and clotting pathways to promote VTE and multi-organ failure (MOF) [11–14].

Although VTE is recognized as a significant problem post-burn injury and post-surgery, the actual prevalence of VTE in burn patients is underreported and there are few prospective studies in thermal injury. The American National Burn Repository describes the risk of VTE in burn patients admitted to intensive care unit as 1.2%, but other studies have suggested that the risk is as high as 22% [15–18]. Heparin resistance has been defined as a heparin requirement greater than 35 000 IU per day, in order to prolong the activated partial thromboplastin time (APTT) into a normal therapeutic range [19]. This definition is not appropriate in burn patients who often present with raised clotting factor VIII and fibrinogen levels that can lead to a reduction in APTT. In order to monitor low molecular-weight heparin therapy more specifically, the anti-factor Xa (AFXa) assay, which leads to a more direct assessment of coagulation is used [19–21]. Typically, the literature suggests that an AFXa level > 0.2 IU/mL defines adequate prophylactic levels [22, 23]. The result of the AFXa assay is a measurement inversely proportional to factor Xa activity and hence represents the activity of low molecular-weight heparin (LMWH). Ordinarily, this should be proportional to the concentration. However, in cases of heparin resistance, there is an observed disparity between AFXa activity and the administered dose of heparin, meaning the two are not proportional. Indeed, a reduced anticoagulant effect of LMWH and unfractionated heparin (UFH) has been shown in a few case reports [24] and two cohort studies in burns [25, 26], although the mechanism(s) of heparin resistance remain largely unknown.

LMWH is a fractionated form of heparin, offering a more predictable pharmacokinetic profile, and with a decreased risk of heparin-induced thrombocytopenia [27–29]. In the UK, LMWH is commonly used for thromboprophylaxis as a result of its favourable profile compared with UFH. Heparin functions through interactions with anti-thrombin III (ATIII) leading to an activity increase of >1000-fold [30]. Both UFH and LMWH interact with ATIII [31, 32]. ATIII is the principle inhibitor of thrombin activity but it is also capable of inactivating multiple clotting factors including factor Xa. Low ATIII levels have also been reported in burn patients, which may contribute to low AFXa activity [33–35].

Heparin is one the most densely negatively charged natural molecules [36]. The clinical antidote to a UFH overdose is protamine sulfate, which exerts its effect by charge interactions with heparin whereby heparin’s ability to bind ATIII, and therefore its anticoagulant effect, are reduced. NETs are extensively released in both trauma and burns; they contain highly positively charged histones that are proteins which promote packaging of DNA into the nucleus [37]. Charge interactions between histones and heparin could theoretically reduce heparin’s anticoagulant effect and therefore significantly contribute to heparin resistance in burn patients [38]. Indeed, there is now some experimental evidence to support this. Rat models of histone H3-induced organ failure have shown that administration of heparin (UFH or LMWH) reduced organ failure and mortality, perhaps by binding histones and preventing their cytotoxic effects [39]. Also, a non-anticoagulant form of heparin has been proposed as a novel therapy to treat sepsis by neutralizing the toxic effects of histones [40]. Two systematic reviews of randomized control trials have also shown that heparin administration significantly reduces mortality in the context of sepsis through non-anticoagulant effects [41, 42]. The effect of histones is not restricted to UFH alone as they can also exhibit anti-LMWH activity [38, 43, 44]. However, this phenomenon has been extensively observed *in vitro*, where most investigators have used purified histones, an approach that is unlikely to reflect the *in vivo* scenario where histones are complexed within short coils of DNA, otherwise known as nucleosomes [45].

We hypothesized that high levels of nucleosomes derived from the degradation of circulating chromatin, for example from NETs, could neutralize polyanionic heparin and therefore contribute to heparin resistance in burn patients. To achieve this, we prospectively measured nucleosome levels, AFXa and ATIII activity in a cohort of severe burn patients.

## Methods

### Participants

Adult patients with a burn size of ≥15% total body surface area (TBSA) were recruited within 24 h of injury into the Scientific Investigation of the Biological Pathways Following Thermal Injury-2 (SIFTI-2) study, a multi-centre prospective cohort study that began in December 2016. In addition, patients were only included if they were on a regular subcutaneous LMWH dosing regimen. Patients were excluded from further analysis if: they were diagnosed with acute kidney injury during, or in the 5 days prior to, sampling time points; if they had a diagnosis of chronic kidney disease; and, if they were taking other anticoagulant medications pre-admission or during sampling time points (for example, direct oral anticoagulants, warfarin, UFH).

Blood samples were collected, 3–5 h after LMWH (i.e. enoxaparin or tinzaparin) dosing on days 5, 10 and 14 post-burn injury. LMWH concentration reaches its maximal peak at this point, tinzaparin a little later than enoxaparin [22, 46–48].

Clinical information collected from participants included: age, sex, percentage TBSA (TBSA%), percentage full-thickness burn (FT%), r-Baux score, LMWH dose prescribed, time from initial burn injury, confirmation of thrombosis (within 90 days of burn admission), Denver MOF and Sequential Organ Failure Assessment (SOFA) scores, death and sepsis (ABA criteria). A confirmed thrombosis was defined as any of the following: deep vein thrombosis confirmed radiologically with Doppler ultrasound scan; pulmonary embolism (PE) confirmed radiologically via CT pulmonary angiogram or ventilation-perfusion scan; indwelling vascular line associated thrombus confirmed radiologically with contrast CT. Routine scanning for asymptomatic thrombosis was not undertaken.

Management of acutely burned patients remained similar throughout the study period. Patients with major burn injury with >15% TBSA were resuscitated as per Parkland’s formula (4 mL per kg per %TBSA), although in cases where there was a history of cardiovascular disease, fluid resuscitation was initiated at 2 mL per kg per %TBSA. Surgical management, if indicated, involved excision and grafting of deep dermal and full-thickness burns within 7 days of injury. Patients with deep dermal or full-thickness burns who were deemed unsuitable for surgical excision were treated with daily topical application of silver sulfadiazine/cerium nitrate. This approach was continued until the patient was optimized for excisional surgery. With regards to the protocol of LMWH use in severely burned patients at the centre, all severely injured patients were started on twice daily dosing (b.d.) 40 mg of enoxaparin (or the equivalent of tinzaparin) unless there was a clinical judgement against this (for example, at extremes of body weight). This regime of high-intensity VTE prophylaxis is in place due to the historical high frequency of breakthrough VTE on standard once daily prophylactic LMWH. It is intended in the protocol that clinical AFXa measurements are taken after the third dose with subsequent adjustments to dosing. If dose is unchanged and AFXa is within a prophylactic range, then AFXa should be taken weekly. However, a recent audit of practice at our centre showed that this was often not followed, likely due to multifaceted reasons including the benign stance on LMWH taken on the ward as well as the difficulty in capturing blood in a particular time-window post-LMWH dose [49]. As a result of this, the study reflects a real-world capture of LMWH dosing. No patients received exogenous ATIII. Equivalence of dosing with different LMWH drugs is recognized and allows us to combine enoxaparin and tinzaparin in analyses with their equivalent doses in international units (IU) of heparin [50].

Healthy controls, aged 18–30, with no comorbidities and receiving no anticoagulant treatment were recruited to compare measurements with samples from clinical burn patients.

### Sample preparation

Peripheral venous blood was collected into a BD Vacutainer™ (BD, Oxford, UK) with 1/10 volume of 3.2% trisodium citrate on days 5, 10 and 14 post-burn injury. Samples were centrifuged twice (2000 × g for 20 min and 13 000 × g for 2 min), the first to generate platelet poor plasma (supernatant), which was subjected to the second spin. Platelet-free plasma was carefully removed and stored at −80°C. Samples were thawed at 37°C for 10 min and mixed prior to analysis.

### Nucleosome measurements

Nucleosomes were measured using a Cell Death Detection ELISA kit v14 following manufacturer’s instructions (Roche; Basel, Switzerland). This ELISA detects nucleosomes (fragmented or whole) by an anti-histone antibody (against H1, H2A, H2B, H3 and H4). Nucleosome levels were quantified as the fold change in absorbance units (AU) when compared with healthy controls. Background and positive controls were measured between assays to control for inter-ELISA variability.

### Cell free DNA (cfDNA)

cfDNA in 10 μL platelet-free plasma was measured using an in-house assay, as previously described [14]. Briefly, 10 μL of plasma samples from healthy controls or burn patients were added to black opaque Corning 96-well plates in duplicate (Thermo Fisher; Massachusetts, USA) and incubated with 140 μL of SYTOX™ green dye (Thermo Fisher) at a working concentration of 1 μM (diluted in PBS) for 10 min in the dark. For calibration a DNA standard curve (0–1000 ng/mL) of λ-DNA (Thermo Fisher) was included in each assay. Healthy control plasma was added to each plate as a negative control. All samples were run in duplicate. Fluorescence was measured using a BioTek Synergy 2 fluorometric plate reader (NorthStar Scientific Ltd; Potton, UK) with excitation and emission wavelengths set at 485 nm and 528 nm, respectively.

### Anti-Xa and ATIII measurement

AFXa and ATIII activity were measured using a Sysmex CS-2100i clotting analyser [51, 52]. Biophen-Hyphen reagents (Biophen, Neuville-sur-Oise, France) were used for calibrator, control and plasma controls. Internal quality control of all assays were performed to ensure correct calibration and assay accuracy.

AFXa levels were measured using the Biophen Heparin LRT chromogenic assay (Ref No: 221013, Lot No: F1801686P3), which is a kinetic method based on the inhibition by ATIII of factor Xa (FXa). The remaining FXa is then measured by its amidolytic activity on a FXa specific chromogenic substrate, which releases p-nitroaniline (pNA). The amount of pNA generated is inversely proportional to the concentration of UFH or LMWH in the tested plasma. This is suitable for heparin, heparin analogues and other direct FXa inhibitors.

ATIII activity was measured using the Biophen AT anti-(h)-Xa LRT assay (Ref No: 221123, Lot No: F1900074) based on the inhibition of FXa by anti-thrombin in presence of heparin. The remaining FXa is then measured by its amidolytic activity on a FXa-specific chromogenic substrate, which releases pNA. The amount of pNA generated is inversely proportional to the ATIII concentration present in the tested plasma. The assay is insensitive to heparin, therefore plasmas from patients on heparin therapy can be tested.

### Statistical analysis

Statistical analysis was conducted using ‘*R*’ 4.0.3 (2020-10-10) [53]. Plots were created using the ‘*ggplot2*’ and ‘*ggpubr*’ packages [54, 55]. Demographic tables were prepared using ‘*tableone*’ [56]. Data preparation involved removal of outlier values in the measured parameters (cfDNA, AFXa, nucleosomes, and ATIII), we used the interquartile range (IQR) method to identify potential outliers, then inspected each value to determine if it was an outlier. This reduced 33 to 30 patients and 55 samples to 49 samples, which were used throughout the analysis.

After removal of outliers, data was checked for normality using the Shapiro–Wilk test and visual inspection of Q-Q plots. Summaries are reported as means and standard deviations or medians and interquartile ranges accordingly. Comparisons between two groups for Normally distributed continuous variables utilized a Student’s *t*-test and for non-Normally distributed data, Wilcoxon testing was used. For a continuous outcome that was modelled by a categorical variable consisting of more than two levels with a Normally distributed continuous variable ANOVA was used, for non-Normal Kruskal-Wallis testing was used. Multiple comparisons correction for the number of comparisons was conducted using a Benjamini-Hochberg procedure. The correlation between two continuous variables was reported using Pearson’s correlation coefficient, *r*.

For the final analysis, univariate association with AFXa of pertinent measured variables was undertaken. Any variables with a statistically significant *p* value (*p* ≤ 0.05) were included in a multivariable model. In addition, several variables that did not meet significance in the univariate round but were thought to be clinically relevant, were included in the multivariable model. The final model, was a linear mixed-effects model implemented in the ‘*lme4*’ [57] package, with scaling of variables using ‘*standardize*’ [58]. To account for intra-participant repeated measurements of samples over the time points, a participant was defined as a factor and included as a random intercept.

Statistical significance was set at a *p* value of ≤0.05. In cases of multiple comparisons, Benjamini-Hochberg False discovery rate (BH-FDR) proceedure was applied.

### Ethical approval

The Scientific Investigation of the Biological Pathways Following Thermal Injury in Adults and Children (SIFTI-2) study was granted ethical approval by Coventry and Warwickshire Research Ethics Committee (reference 16/WM/0217). ﻿Where possible, written informed consent was received from participants before their inclusion in the study. However, due to the severe nature of the injuries, the ethics committee approved the use of a legal consultee, either personal or nominated, if the patient was not initially able to consent for inclusion in the study themselves. When the patient regained capacity, they were approached to give written consent to continue to participate in the study or could completely withdraw.

## Results

### Patient demographics and sampling time points

Thirty participants were included in this prospective observational cohort study (Table 1). All injuries were either flame or flash burns. Mean TBSA was 44% (SD 16.7) with an r-Baux score of 98 (SD 26). Two patients died within 30 days of injury. Patients received either enoxaparin (16, 53%) or tinzaparin (14, 46%) as LMWH prophylaxis. Due to issues with dose timing, not every patient had a sample taken at each time point. The rates of adherence with the sampling schedule were 19 (63%), 14 (47%) and 16 (53%) on days 5, 10 and 14, respectively. Average time from LMWH administration to sample being drawn was 03:18 (hh:mm) (IQR 03:07–03:42). Of 49 samples, 48 (98%) were taken within 3 to 5 h post-LMWH. Only one patient had a confirmed VTE, which was a bilateral PE identified on CTPA.

**Table 1:** Patient demographics

**Variables**	**Value**
Patients, n	30
LMWH drug received	
Enoxaparin	16 (53.3)
Tinzaparin	14 (46.7)
Age, mean (SD)	45.07 (15.22)
Sex, male (%)	24 (80.0)
BMI, median (IQR)	25.50 (23.25, 30.40)
Injury mechanism (%)	
Flame	29 (96.7)
Flash	1 (3.3)
TBSA%, mean (SD)	43.98 (16.70)
FT%, mean (SD)	27.47 (21.08)
Inhalation injury (%)	
No	13 (43.3)
Yes	17 (56.7)
r-Baux, mean (SD)	97.93 (25.98)
ABSI, mean (SD)	8.30 (2.95)
Denver MOF, median (IQR)	3.00 (2.00, 4.00)
SOFA, median (IQR)	11.00 (7.00, 12.00)
Confirmed VTE (%)	1 (3.33)
Sepsis (%)	20 (66.7)
Time to sepsis (ABA criteria) (days), median (IQR)	3.50 (2.00, 5.25)
Death (%)	4 (13.33)
Death within 30 days (%)	2 (6.67)
Time to death (days), median (IQR)	22.00 (11.50, 58.00)

Represented as either total number (%) or, after testing for normality, as median (IQR) or mean (SD)

*LMWH* low molecular-weight heparin, *BMI* body mass index, *TBSA* total body surface area, *FT%* percentage of full-thickness burns, *ABSI* abbreviated burn severity index, *MOF* multi-organ failure, *SOFA* sequential organ failure assessment score (on admission), *VTE* venous thromboembolism, *IQR* interquartile range, *SD* standard deviation

### Incidence of heparin resistance

Thirty-one of 49 samples (63%) displayed a peak AFXa level below 0.2 IU/mL and hence were defined as heparin resistant. Of the 30 patients, 23 (77%) displayed heparin resistance at some stage during sampling in the first 2 weeks post-injury. The overall mean peak AFXa level was 0.18 (SD 0.11).

### The effect of increasing doses of heparin on anti-factor Xa activity

**Figure 1.**
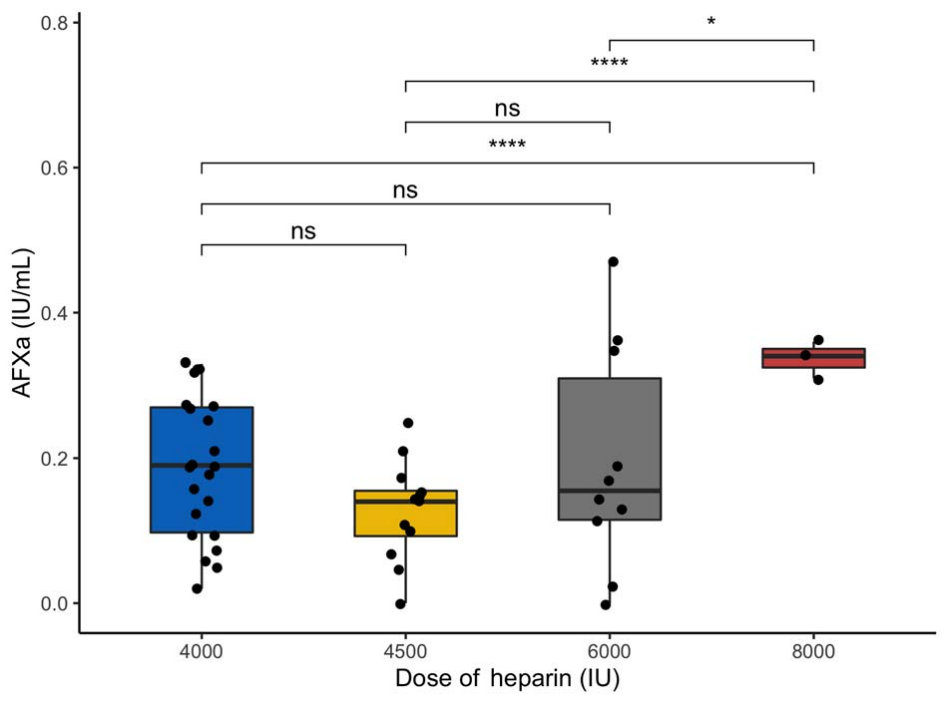
Peak anti-factor Xa (AFXa) level increases with increasing LMWH dose. Points represent individual samples taken at any of the time points (days 5, 10 and 14) at peak LMWH dose (3–5 h post-dose). Heparin dose 3500 IU was removed as there was only one observation at this value. At 4000 IU median AFXa = 0.19 (0.09–0.27), 4500 IU 0.14 (0.09–0.16), 6000 IU 0.16 (0.11–0.35), 8000 IU 0.34 (0.31–0.36). ANOVA is significant *p* = 0.0274. Asterisk represents multiple-comparison corrected *p* values (B-H FDR) ^*^p < 0.05, ^*^^*^p < 0.01, ^*^^*^^*^p < 0.001, ^*^^*^^*^^*^p < 0.0001. *FDR* false detection rate, *ns* non-significant, *LMWH* low molecular-weight heparin

Analysis of the 49 samples by LMWH dose administered prior showed peak AFXa levels were 0.19 IU/mL (IQR 0.09–0.27) at 4000 IU, 0.13 IU/mL (0.08–0.15) at 4500 IU, 0.17 IU/mL (0.12–0.32) at 6000 IU, and 0.34 IU/ml (0.32–0.36) at 8000 IU (Figure 1).

**Figure 2.**
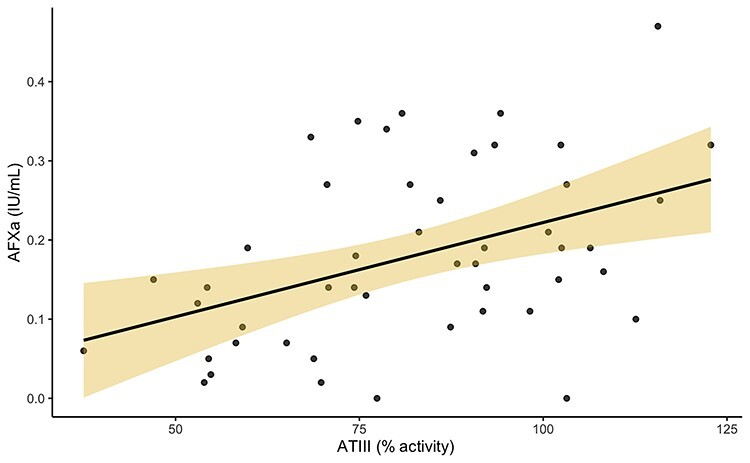
Correlation between peak AFXa and ATIII levels. Peak AFXa levels as explained by ATIII (% activity), points are plotted in grey and represent individual samples taken across all time points on day 5, 10 and 14. Solid black line represents the linear regression fitted to the data, with shaded region of 95% CI. *r* = 0.43, *p* = 0.0021. *ATIII* anti-thrombin III, *AFXa* anti-factor Xa

### Levels of ATIII in burn patients receiving heparin therapy

The normal range for ATIII activity is 80–120%. The mean ATIII level across all 49 samples, 30 patients was 81.93% (SD 20.38) with a minimum value of 37.5%, and maximum activity of 122.8%. Levels of ATIII activity were below 80% in 23 samples (47%), which corresponds to 17 participants (57%) at some point in the first 2 weeks of admission. Figure 2 shows the correlation of peak AFXa level with ATIII levels, *r* = 0.43 (*p* = 0.0021).

### Levels of nucleosomes

Nucleosome levels were higher in burn patients (n = 30) (AU) [median = 0.2 AU (IQR 0.1–0.3)] when compared with samples from healthy controls (n = 6), 0.03 AU (IQR 0.02–0.05), representing a median fold increase of 6.75 AU (IQR 3.5–10.04) (*p* < 0.001).

### Changes in nucleosome, cfDNA, anti-factor Xa and ATIII over study period

**Figure 3.**
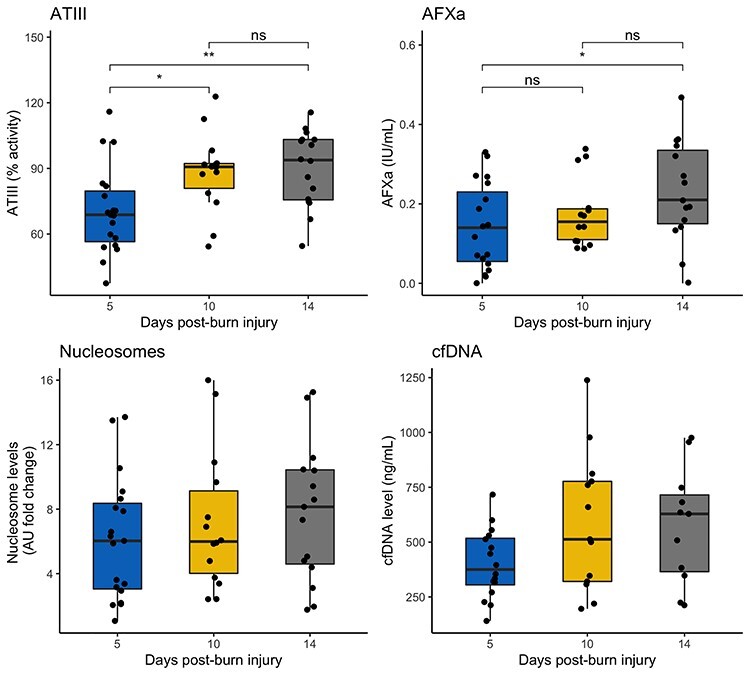
Facets showing variable levels at all sampling time points of day 5, 10 and 14. Points represent samples. With ANOVA or Kruskal-Wallis (depending on normality). Nucleosome levels (ANOVA *p* = 0.24) and cfDNA (Kruskal-Wallis *p* = 0.055) demonstrated no change over time. ATIII (ANOVA *p* = 0.002) and peak AFXa (ANOVA *p* = 0.026) did demonstrate change over time. Asterisk represents multiple-comparison corrected *p* values (B-H FDR) ^*^p < 0.05, ^*^^*^p < 0.01. *ATIII* anti-thrombin III, *AFXa* anti-factor Xa, *cfDNA* cell-free DNA, *FDR* false detection rate, *ns* non-significant

**Figure 4.**
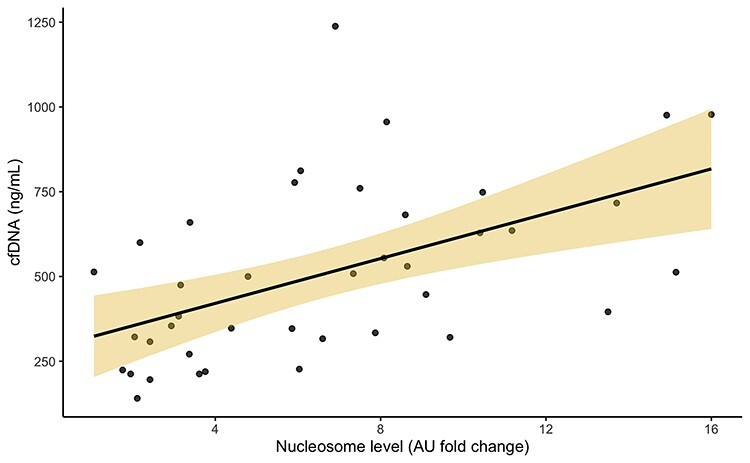
Correlation between cfDNA and nucleosome levels. cfDNA levels and nucleosome (AU fold change from healthy control) are shown as grey points (samples). The solid black line shows the linear regression fitted to the data, with the shaded region 95% CI. *r* = 0.57, *p* = 0.0001. *cfDNA* cell-free DNA, *AU* absorbance units, *CI* confidence interval

Across time (Figure 3), nucleosome levels showed no statistically significant change between day 5 [6.03 AU (IQR 2.99–8.50)], day 10 [5.99 AU (3.76–9.68)], and day 14 [8.15 AU (4.49–10.45)], *p* = 0.24. Neither did cfDNA levels with median values of 375 ng/mL (IQR 294–522) on day 5, 513 ng/mL (317–786) on day 10, and 629 ng/mL (356–732) on day 14, *p* = 0.055. AFXa showed a significant increase between day 5 [0.14 IU/mL (0.05–0.24)] and day 14 [0.21 IU/mL (0.15–0.34)], ANOVA *p* = 0.026. ATIII levels showed an increase from day 5 [68.8% (55.7–80.8)] to day 10 [90.7% (78.7–92.3)] and day 14 [93.8% (75.4–103.2)], ANOVA *p* = 0.002. Nucleosome levels were shown to correlate positively with cfDNA levels *r* = 0.57 (*p* = 0.0001) (Figure 4).

**Table 2:** Parameters stratified by sepsis

**Variables**	**No sepsis**	**Sepsis**	***P* value**
**Patients, n**	10	20	
Heparin resistance			
Yes	5 (50)	15 (75)	ns
No	5 (50)	5 (25)	
Age, mean (SD)	40.90 (12.87)	47.15 (16.16)	ns
Sex, Male (%)	7 (70)	17 (85)	ns
BMI, median (IQR)	23.5 (22, 32.5)	27.12 (24.92, 29.46)	ns
TBSA%, mean (SD)	37.7 (14.98)	47.12 (16.97)	ns
FT%, mean (SD)	12.85 (17.73)	34.77 (18.99)	0.005
r-Baux, mean (SD)	83.6 (23.61)	105.1 (24.57)	0.03
ABSI, mean (SD)	7.1 (2.81)	8.9 (2.9)	ns
Denver MOF, median (IQR)	0 (0, 2)	4 (3, 4)	<0.001
SOFA, median (IQR)	4 (1, 6)	11 (11, 12)	<0.001
**Samples, n**	14	35	
cfDNA (ng/ml), median (IQR)	267.28 (217.70, 386.09)	577.38 (347.20, 751.33)	0.001
Nucleosomes (AU fold change), mean (SD)	5.17 (4.12)	7.7 (3.89)	0.049
ATIII (% activity), mean (SD)	95.99 (16.58)	76.30 (19.16)	0.002
AFXa (IU/mL), mean (SD)	0.22 (0.12)	0.16 (0.11)	0.142

Represented as either total number (%) or, after testing for normality, as median (IQR) or mean (SD). Statistical testing with Fisher's exact test for parametric variables, and Kruskal-Wallis test for non-parametric variables

*ns* not significant, *BMI* body mass index, *TBSA* total body surface area, *FT%* percentage of full-thickness burns, *ABSI* abbreviated burn severity index, *MOF* multi-organ failure, *SOFA* sequential organ failure assessment score (on admission), *ATIII* anti-thrombin III, *AFXa* anti-factor Xa, *IQR* interquartile range, *SD* standard deviation

**Figure 5.**
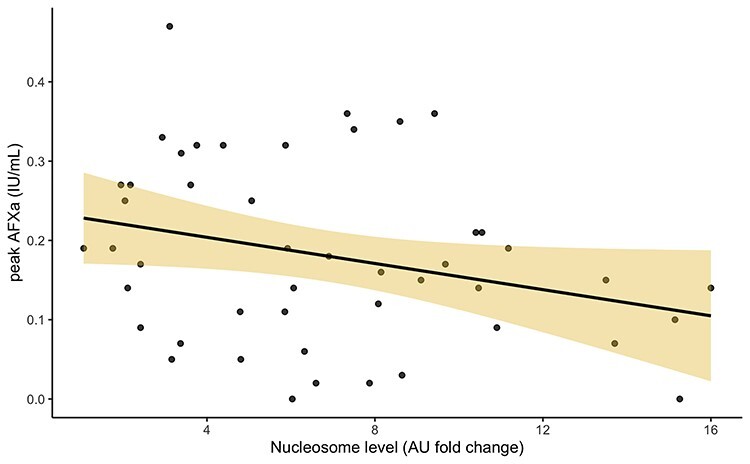
Correlation between peak AFXa and nucleosome levels. Peak AFXa level as explained by the nucleosome AU fold change from healthy control. Each point represents a sample. Taken over all time points, days 5, 10 and 14. A linear regression model fitted and is shown by the black line. Shaded area shows 95% CI. *r* = −0.29, *p* = 0.050. *AFXa* anti-factor Xa, *AU* absorbance units, *CI* confidence interval

### Differences between measures in sepsis groups

Twenty patients (66.7%) developed sepsis during their inpatient stay. This provided an opportunity to consider differences in the measured parameters (Table 2). Most parameters relating to injury severity were significantly higher in sepsis patients. cfDNA was significantly higher 577 ng/mL (IQR 347–751) in patients with sepsis compared with non-septic patients 267 ng/mL (IQR 218–386) (*p* = 0.001). Nucleosomes showed the same increase, 7.7 AU fold change (SD 3.89) *vs* 5.17 AU fold change (SD 4.12), respectively, *p* = 0.049. ATIII was significantly lower in septic patients, 76.3% activity (SD 19.2) *vs* non-septic 96.0% activity (SD 16.6), *p* = 0.002. Peak AFXa was not significantly different between the groups, but was slightly lower in samples taken from septic patients, 0.16 IU/mL (SD 0.11), *vs* samples taken from non-septic patients, 0.22 IU/mL (SD 0.12), *p* > 0.05.

### Heparin resistance and associated measures

Before moving on to consider the factors affecting AFXa levels, we compared variables stratified by the dichotomous outcome, heparin resistance (defined as AFXa < 0.2 in a sample). There were no significant differences between demographic variables in patients who displayed heparin resistance (n = 20) *vs* no heparin resistance (n = 10). There was a mild decrease in ATIII activity in heparin-resistant samples [76.6% activity (SD 20.6)] *vs* non-resistance [91.4% activity (SD 16.3)], *p* = 0.011. There was a non-significant mild increase in nucleosomes in resistant samples [7.81 AU fold change (SD 4.39)] *vs* non-resistance [5.42 AU fold change (SD 2.98)], *p* = 0.051. There was no association for cfDNA between resistant [500 ng/mL (IQR 319–733)] and non-resistant samples [383 ng/mL (IQR 322–629)], *p* = 0.52.

### Association between nucleosomes and anti-factor Xa levels

The correlation between peak AFXa and nucleosome levels showed a negative direction (Figure 5), *r* = −0.29, *p* = 0.050. With this in mind, we approached model creation to assess the relationship between peak AFXa and nucleosomes when several confounders are adjusted for. The first step tested: age, TBSA, ATIII activity, cfDNA, nucleosome levels, LMWH dose given pre-sample, day sample taken, drug given (tinzaparin or enoxaparin), and sepsis. Significant factors included ATIII activity (*p* = 0.0021), LMWH dose given pre-sample (*p* = 0.028), day sample taken (*p* = 0.026), and nucleosomes (*p* = 0.050).

The final model included those statistically significant variables as well as variables considered clinically important. AFXa was the response variable and independent variables included: nucleosome levels, ATIII activity, TBSA%, LMWH dose given pre-sample, day sample taken, sex, age, drug given (tinzaparin or enoxaparin). Participant was included as a random intercept term to account for multiple measurements made on the same participants. This model (overall R^2 = 0.44) was clearly significant for ATIII (*p* = 0.0053), LMWH dose pre-sample (*p* = 0.0049), drug given (tinzaparin or enoxaparin) (*p* = 0.03), and nucleosome levels (*p* = 0.0453) (Table 3).

**Table 3:** Multivariable model output, AFXa as the response variable, with 95% confidence intervals

**Variables**	**Effect estimate**	**95% confidence interval**	***P* value**
Nucleosomes	−0.25	−0.51 to −0.007	0.045
ATIII	0.45	0.14 to 0.75	0.005
LMWH dose	0.36	0.12 to 0.62	0.005
Drug, enoxaparin	0.32	0.03 to 0.60	0.03
%TBSA	0.05	−0.22 to 0.32	0.69
Gender, female	0.05	−0.23 to 0.34	0.70
Age	0.14	−0.12 to 0.40	0.27
Time point	0.013	−0.33 to 0.36	0.94

Input scaled to allow comparison

*ATIII* anti-thrombin III, *LMWH* low molecular weight heparin, *TBSA* total body surface area, *AFXa* anti-factor Xa

## Discussion

This prospective study set out to identify and study potential mechanisms of heparin resistance in a cohort of burn patients.

Our results demonstrate that a significant proportion of patients [23 of 30 (76.7%)] were heparin resistant (i.e. below an AFXa activity of 0.2) within the first 2 weeks of injury. This was despite at least twice-daily LMWH dosing. Although only 1 patient in our cohort experienced a clinically apparent VTE event, Levine *et al*. showed that in their cohort of patients, post hip replacement, 14.9% patients with an AFXa of <0.1 had a thrombotic event [59]. Our study did not utilize regular imaging, such as Doppler ultrasound of the lower limb to detect subclinical thrombosis.

Although the overall median ATIII level was normal, 17 participants (56.7%) displayed low ATIII levels at some point in the first 2 weeks post-burn. A deficiency in ATIII would contribute to decreased AFXa activity *in vivo* despite large doses of LMWH. ATIII levels correlated with peak AFXa levels (*r* = 0.43, *p* = 0.0021). The assay used relies on at least some baseline activity of endogenous AT, and may not truly reflect all the variables associated with optimal FXa inhibition *in vivo*. ATIII deficiency has previously been described in burn injury [34, 35, 60], and replacement therapy has even been suggested to maintain levels and optimal heparin anticoagulation [35]. Most (96%) of our samples had at least 50% ATIII activity. It is unlikely at these levels that the AFXa assay would have been affected. Regardless, we included ATIII activity as a covariate in the multivariable regression analysis between AFXa and nucleosome levels to adjust for any potential confounding effects.

Nucleosome levels [6.75 AU, (IQR 3.5–10.04) times normal] were raised significantly compared with healthy control levels in this cohort of severe burn patients. As one might expect, nucleosomes and cfDNA levels also correlated as they would be released either by cell necrosis during injury and/or from neutrophils via NET formation [14]. Time relationship of the variables were studied to ensure this would not have a confounding effect on further analysis. Nucleosome levels and cfDNA levels demonstrated no change over the three sampling time points, but for cfDNA there was nearly an association (*p* = 0.055) [14]. ATIII was increased significantly between days 5, 10 and 14 (*p* = 0.002) suggesting an element of recovery. AFXa also showed an increase between days 5 and 14 (*p* = 0.026). Six patients received dose increases as part of their clinical care, as per the protocol described in Methods – Participants, which may explain this increase. As shown increases in LMWH dose led to significantly higher levels of AFXa, and high doses (8000 IU) were able to overcome heparin resistance. These data suggest the interference of heparin’s action is in some way competitive with heparin, or affecting heparin itself. Samples from participants collected during sepsis showed significantly elevated levels of cfDNA, which is in agreement with previous evidence [14]. Nucleosomes were also elevated in samples collected during sepsis, which is in concert with the hypothesis that a potential source of nucleosomes is NETosis [14, 61].

Samples displaying heparin resistance showed a decrease in ATIII activity, and a non-significant increase in nucleosomes (*p* = 0.051). However, dichotomizing our analysis using a peak AFXa cut-off of 0.2 defining heparin resistance, leads to unnecessary loss of power, and instead we chose to understand the effect of nucleosomes on peak AFXa levels directly. Assessing the correlation in samples between circulating nucleosomes and peak AFXa levels (Figure 5) found a significant correlation, Pearson’s *r* = −0.29 (*p* = 0.050). This negative correlation supports our hypothesis that an increase in nucleosomes would interact with heparin and thus reduce AFXa activity, a measure of heparin’s biological activity. To understand the effect of nucleosomes on AFXa we created a model, independent of several confounders and correcting for repeated measurements with a random effect. The univariate models for several variables showed that ATIII, LMWH dose pre-sample, and day sample taken were significantly associated with AFXa. These were taken forward into the multivariate model, along with clinical factors estimated to be important in this interaction. Nucleosomes gained further significance in this model (*p* = 0.0453) suggesting that they have a significant impact on peak AFXa levels even when correcting for several confounding variables.

This corresponds with *in vitro* data studying the effects of calf-thymus histones on heparin’s anticoagulant activity using calibrated automated thrombography (CAT) [44]. Although there is scepticism regarding *in vitro* observations, as pure calf-thymus histones, with no associated DNA, is an unrealistic physiological state. Immunofluorescence data suggests that heparin still has the ability to bind histones complexed with DNA in NETs, and this supports the analysis conducted in these burn patients [62]. In addition, recent papers have focused on the application of heparin to attenuate nucleosome-induced inflammatory responses, which is the alternate perspective of our hypothesis [40, 63]. These profound *in vitro* effects of histones on heparin may be reduced or affected *in vivo* by flow dynamics. Of interest, data have also shown that when NETs are released within a blood vessel, DNase will effectively release the DNA into circulation but histones remain adhered to the vessel endothelium, likely due to their charge [64]. Heparin has also been shown to dismantle NETs [13]. Clearly, the interactions *in vivo* are complex but also apparent in our analysis of these severely burn-injured patients.

Other potential effects on heparin resistance involve the bioavailability of LMWH. In the initial stages after burn trauma, aggressive fluid resuscitation as part of medical treatment leads to an increase in the volume of distribution [65, 66]. This could contribute to reduced heparin concentrations and reduced anticoagulant activity. During the hypermetabolic state of burn injury, renal clearance can also be altered [67]. UFH is primarily removed by the reticuloendothelial system with very minor renal excretion [68]. Enoxaparin, in contrast, has around 40% renal excretion and hence may have a reduced half-life [69]. However, inter-patient variability in renal clearance of drugs is high and therefore it would be surprising to see this effect reliably, as renal clearance may also be reduced leading to an increase in heparin concentration [70]. In addition, it would be expected that these variables would relate to burn severity and TBSA, which we included in the multivariate analysis for adjustment.

A final explanation for heparin resistance may involve other heparin binding proteins. Examples include platelet factor 4 (PF4) and factor VIII. Factor VIII causes an apparent heparin resistance; however, this requires extremely high and rarely seen, even in burns, concentrations of FVIII [20]. PF4 is a cationic protein released from the alpha-granules of activated platelets. *In vitro* it has been shown to have anti-heparin activity for unfractionated and LMWH affecting both their anti-thrombin and AFXa activities [71, 72]. Hence, it may be released by platelets in the acute inflammatory response of burn injury and also bind to and potentially impair heparin biological activity. Similarly to FVIII, Baglin *et al*. reported that LMWH was subject to less interference from PF4 with respect to anticoagulant activity [73] and very high levels would need to be achieved to produce the profound effect seen on peak AFXa levels in this cohort.

### Study limitations

Peak AFXa data was limited due to a number of samples being taken outside the 3–5 h post-dose window. Hence, compliance with the protocol could have been improved, but actually mirrors the issues seen in clinical practice with timing of AFXa collection and changes to dose regimen [49]. Some studies report 3–5 h as the peak LWMH activity time; however, we enabled a slightly larger window to account for tinzaparin, which represented approximately half of our LMWH samples and has a slightly later peak activity. In addition, only 1 of the 30 patients included exhibited a VTE event, which meant analysis with VTE outcomes could not be performed.

Clinical considerations as a result of this paper should focus on implementation of an accurate blood draw sampling of peak AFXa after LMWH dosing as well as clear guidelines on therapy increments, potentially utilizing reminders through ward-based electronic systems. There is some argument that the standard baseline LMWH dose given to severe burn-injured patients should increase; however, centres must take into consideration the increased risks of bleeding, as well as potentially heparin-induced thrombocytopenia, associated with this and implications around operative procedures. Further evidence is required to derive this. Our results show that recombinant exogenous ATIII therapy may be beneficial to promote heparin’s anticoagulant action and overcome resistance, although increasing the heparin dose itself may have other currently unperceived benefits on the alternative perspective of this work, which is neutralizing cytotoxic nucleosomes. Lastly, this is another contribution of evidence that measuring NETosis in burn patients clinically could guide therapy on the coagulation aspect of burn care.

## Conclusions

A significant proportion of patients with severe burn injury show heparin resistance [23 of 30 (77%)] for the first 2 weeks post-burn injury in this study. Nucleosome levels are elevated significantly in burn patients, likely as a result of tissue injury and NET release. Nucleosome levels correlated significantly with AFXa, and in a multivariable mixed-effects model adjusting for potential confounders-including ATIII activity shown to be reduced in severe burn injury-remained significantly associated with AFXa. The negative association between nucleosomes and peak AFXa levels supports the hypothesis that nucleosomes are affecting the anticoagulant ability of heparin.

## Abbreviations

AFXa, anti-factor Xa; APTT, activated partial thromboplastin time; ATIII, anti-thrombin III; AU, absorbance units; b.d., twice daily dosing; CAT, calibrated automated thrombography; cfDNA, cell-free DNA; FT%, percentage full-thickness burn; h, hour; IQR, interquartile range; LMWH, low molecular-weight heparin; MOF, multi-organ failure; NETs, neutrophil extracellular traps; PE, pulmonary embolism; PF4, platelet factor 4; pNA, p-nitroaniline; TBSA, total body surface area; UFH, unfractionated heparin.
